# Encapturing triclosan from water using a novel sonoadsorbent triazine polymer with carbohydrazide linkages: experimental and theoretical studies

**DOI:** 10.1039/d5ra02743h

**Published:** 2025-09-15

**Authors:** Silpa Elizabeth Peter, Saumya Krishnan, C. V. Suneesh, Paul Thomas, Jayasree Elambalassery, P. Vairavel, N. V. Anil Kumar

**Affiliations:** a Department of Chemistry, Manipal Institute of Technology, Manipal Academy of Higher Education Manipal – 576104 India; b Department of Chemistry, University of Kerala Kariavattom Campus Thiruvananthapuram Kerala India suneesh@keralauniversity.ac.in; c Department of Physics and Technology, University of Bergen Norway paul.thomas@uib.no; d Department of Applied Chemistry, Cochin University of Science and Technology Kochi – 682022 Kerala India jelambal@cusat.ac.in; e Department of Chemical Engineering, Manipal Institute of Technology, Manipal Academy of Higher Education Manipal – 576104 India

## Abstract

Triclosan (TCS), an active ingredient in personal hygiene products, has been considerably used and found in water bodies due to its increasing consumption. Even at minimal concentrations, TCS can harm living organisms, contributing to antimicrobial resistance and disrupting the reproductive and endocrine systems, raising significant environmental and health concerns. In this study, we developed a novel triazine-rich porous polymer, CCCH CTP (from cyanuric chloride and carbohydrazide), which exhibits a semicrystalline structure with spherical morphology. It is characterized by a porous area of 24 m^2^ g^−1^ with thermal stability up to 250 °C. It is used as an adsorbent supported by ultrasonication to remove TCS with an efficiency of 81% in acidic pH 3. The adsorption process reached saturation within 25 minutes, unveiling the effectiveness of the polymer. The maximum adsorbing capacity was determined to be 83.89 mg g^−1^, agreeing in terms of pseudo-second-order kinetics with the Freundlich isotherm model, indicating favorable chemisorption characteristics. These results were validated at the molecular level by the DFT calculations. These findings emphasize the potential of such porous materials in environmental remediation, highlighting their role in tackling the challenge of TCS contamination in water bodies.

## Abstract 2

Application of porous polymers in capturing triclosan, an emerging pollutant, from water.

## Graphical abstract

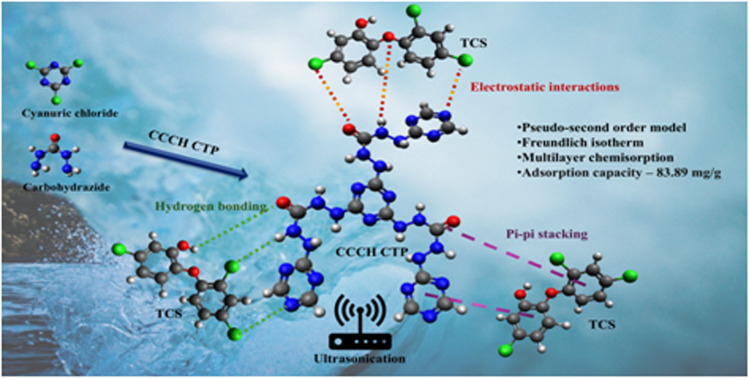

## 1. Introduction

Our planet Earth is covered by 71% water, and only 3% is fresh and suitable for consumption by living organisms.^1^ This limited fresh water is increasingly polluted with various contaminants, from colored dyes (industrial activities) to colorless organic pharmaceuticals that enter water systems as a result of improper disposal.^2^ The rapid industrialization, urbanization, and intensified agricultural activities have caused the pollution of freshwater resources. These contaminants pose serious risks to human health, often exceeding safer limits considered permissible for drinking water. As the global population grows, the demand for clean water prompts us to address the purification of our limited freshwater.

**Fig. 1:**
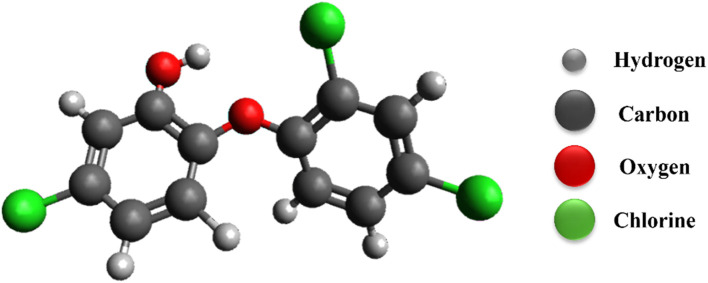
Chemical structure of triclosan.

In recent years, personal care products have gained widespread popularity for their role in enhancing human lifestyle and hygiene.^3^ Among the myriads of components found in these products, triclosan (TCS) – chlorinated aromatic hydroxy ether – stands out for its notable antimicrobial properties, making it a common additive in cosmetics and personal hygiene products (Fig. 1). However, the excessive use of these products has led to critical levels (0.6–2.3 μg L^−1^) of TCS accumulating in water bodies.^4^ The presence of TCS in aquatic environments raises significant concerns due to its potential to foster antimicrobial resistance in microorganisms, posing a severe threat to global health. They are ionizable (which is pH dependent) due to hydroxy functionalization and are toxic in nature. Being lipid soluble (hydrophobic), they can bioaccumulate and magnify along the food chain, creating a further risk to the other living species. As an emerging environmental pollutant, triclosan can disrupt growth and reproductive processes (birth defects in newborn) by interfering with hormonal systems (thyroid, nervous system) and is carcinogenic in nature (liver, kidney, and colon).^5–7^ They are susceptible to photodegradations, producing hazardous intermediates. In order to ensure a better ecosystem for living organisms, these risks highlight the necessity for developing and researching effective methods to remove such pollutants among the existing pollutants (dyes and heavy metals).

Water purification can be achieved through various techniques, including biological methods (using microbes),^8^ chemical processes (such as oxidation, ozonation, and chlorination), and physical methods (adsorption, ion exchange, and membrane filtration).^2,9,10^ Among these, adsorption is considered superior due to its cost-effectiveness, high removal efficiency, and the absence of hazardous intermediates or by-products formed.^11^ Over the years, various porous adsorbents have been utilized, including inorganic (such as silica, clay, and zeolites), organic (like polymers and activated carbon), and hybrid materials (metal–organic frameworks and nanocomposites). However, these materials often face challenges related to chemical stability, structural control, and regeneration capacity, making the search for an effective adsorbent quite difficult.^11^

Porous organic polymers (POPs) are gaining attention for being effective adsorbents owing to their unique properties. These polymers are synthesized from organic linkers comprising lighter elements (C, N, O, S, H), interconnected through covalent bonds. These porous structures provide chemical and thermal stability and result in a low-density, high-surface-area network.^12^ The symmetry of the building blocks influences the geometry of the pores. Being less toxic, we can tune their pore structure (choosing suitable building blocks) along with post-functional modifications to enhance their suitability as adsorbents with large surface area and numerous adsorption sites.^13^ Covalent triazine polymers, synthesized from triazine linkers, can be exploited as adsorbents, as reported in previous literature. They can adsorb dyes (Rhodamine B, Congo red), metals (mercury), antibiotics (tetracycline, sulfonamides), and bisphenols.^14–19^ The abundant nitrogen atoms (having lone pairs of electrons), along with other functional groups in the polymer, promote adsorption through electrostatic, π–π interactions, and hydrogen bonding.^20^

Recently, ultrasound-assisted adsorption has been used in purification techniques.^21,22^ In the presence of highly energetic ultrasounds, ultrasonic cavitation occurs, followed by a series of processes resulting in its collapse, generating high temperature and pressure conditions. It promotes the easy diffusion of pollutants into the adsorbents, thus improving the removal efficiency of the material.^21,22^ Although the practical applications of these materials as a sonoadsorbent are still in the early stages of development, they hold significant promise for improving water purification processes, suggesting a future where clean water may be more accessible.

The current study is focused on analyzing the adsorption capacity of a novel porous polymer, CCCH CTP, synthesized from cyanuric chloride and carbohydrazide, making it an effective adsorbent for TCS *via* ultrasonication. To the best of our knowledge, the literature lacks any prior research on the synthesis of this triazine-based polymer and its applicability in adsorbing TCS from water as a sonoadsorbent. The different mathematical models of kinetic and isotherm studies must be evaluated, describing the dynamics of the adsorption and optimizing the conditions for maximum removal of TCS. This polymer functions efficiently and contributes to reduced energy and operational costs for removing TCS in real-world applications. The presence of aromatic nitrogen-rich triazine structures, along with carbonyl and amino functional groups within the polymer, serves as an essential factor in facilitating the TCS adsorption from aqueous environments.

This research underscores the urgent need to tackle the rising concerns regarding emerging pollutants in water sources, particularly triclosan, which are often detected at harmful levels. Using this triazine polymer, we intend to deepen our understanding of its potential in water purification processes as a sonoadsorbent, adding a valuable contribution to this field. Comparison of this adsorbent with existing materials for triclosan removal further emphasizes the significance and relevance of our study in addressing contemporary water quality challenges.

## 2. Experimental section

### 2.1 Materials

Analytical-grade chemicals and solvents were utilized without any purification for the experiments. Cyanuric chloride, carbohydrazide, 1,4-dioxane, triethyl amine, methanol, tetrahydrofuran, and acetone were purchased from Sigma-Aldrich. The pollutant triclosan (TCS) was purchased from Himedia, India. Distilled water was utilized throughout the adsorption study.

### 2.2 Instrumentations

Fourier transform infrared (FTIR) spectra were recorded using the Attenuated Total Reflectance (ATR) on the Shimadzu 400 MHz Bruker spectrophotometer (range of 400–4000 cm^−1^). The solid-state ^13^C Cross Polarization Magic Angle Spinning Nuclear Magnetic Resonance (CP/MAS NMR) analysis was performed on a Jeol 400 MHz NMR spectrometer. X-Ray Diffraction (XRD) data was analyzed with Rigaku Miniflex 600 (5th generation) X-ray diffractometer. Thermogravimetric analyzer (TGA) assessed the material's thermal stability under a nitrogen atmosphere with temperatures ranging from 30–800 °C at the heating of 10 °C min^−1^. Nitrogen adsorption at 77 K was measured using a Quantachrome Autosorb IQ-MP-XR gas sorption instrument, with analysis conducted *via* ASiQwin software. The Non-Local Density Functional Theory (NL-DFT) method was employed to measure pore size and its distribution. Field-emission scanning electron microscopy (FE-SEM) and energy dispersive spectroscopy (EDS) were performed using Jeol HR-FESEM 7610FPLUS. X-ray photoelectron spectroscopy (XPS) spectrum was analyzed on a Thermofisher Nexsa XPS instrument. The absorbance of triclosan was recorded at 280 nm using a Shimadzu UV-1800 spectrophotometer.

### 2.3 Synthesis of CCCH CTP

**Fig. 2:**
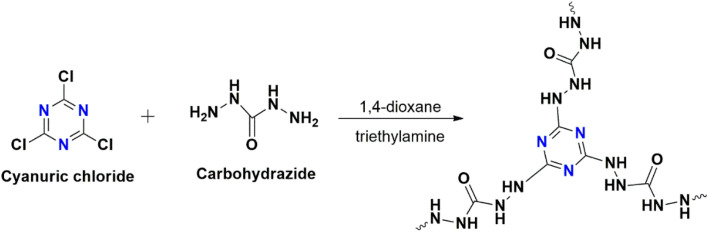
Synthetic protocol for CCCH CTP.

Covalent triazine polymer CCCH CTP was synthesized by modifying the reported protocols^14,23^ based on nucleophilic substitution reaction as in Fig. 2. Initially, carbohydrazide (270 mg, 3 mmol) and triethylamine (0.5 mL, 3.6 mmol) were dissolved in dioxane (15 mL) in a 100 mL round-bottomed flask under an inert nitrogen atmosphere. Cyanuric chloride (368 mg, 2 mmol) dissolved in dioxane (15 mL) in ice-cold conditions was added dropwise to the above solution with stirring at 25 °C. Then, it was refluxed for 48 hours until the polymerization was completed. A pale, yellow-colored precipitate formed was separated (filtration) and washed with dioxane, water, methanol, tetrahydrofuran, and acetone repeatedly (till clear filtrate). The product obtained was purified using Soxhlet extraction from methanol for 24 hours and finally dried in a vacuum (120 °C) to obtain CCCH CTP (yield: 87%). It was then characterized and analyzed using different techniques.

### 2.4 Adsorption studies

#### 2.4.1. Batch adsorption studies

A stock TCS solution (100 ppm) was made by dissolving 50 mg of triclosan in 500 mL absolute ethanol due to its better solubility in ethanol than in water.^12^ It was then stored in a light-protected atmosphere at 4 °C to avoid possible degradation. The required TCS concentrations were prepared by dilution of the stock solution with distilled water and were calibrated, which serves as a standard for determining the concentration of triclosan molecules.

The required polymeric adsorbent (CCCH CTP) was added to the adsorbate (TCS) solution in a 250 mL standard flask. The adsorption was facilitated by sonication using a Labman ultrasound-assisted water bath (100 W power and 40 kHz frequency) for 1 hour at 25 °C. It was then centrifuged at 10 000 rpm (15 minutes). 5 mL of aliquots were taken to measure the absorbance using a UV-visible spectrophotometer of the unadsorbed TCS pollutant at 280 nm. All the experiments were repeated thrice, and the mean absorbance value was considered for the calculations. The adsorption capacity (*q*_e_, in mg g^−1^) and percentage removal of triclosan (*R*, %) were determined using mathematical equations discussed below:1
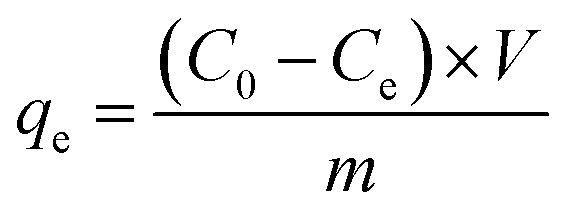
2
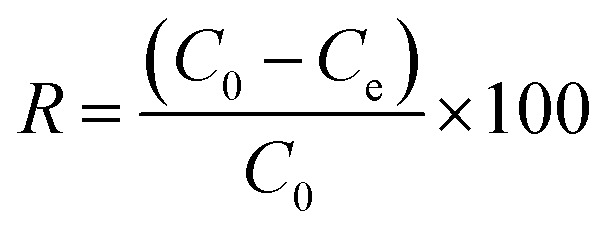
*C*_0_ and *C*_e_ represent the initial and equilibrium TCS concentration, *V* is the TCS volume, and *m* is the mass of CCCH CTP adsorbent.

#### 2.4.2. Optimization of parameters affecting adsorption

The different parameters affecting the TCS adsorption were explored. The pH effect was analyzed in 10 ppm TCS solution (100 mL) with 15 mg of adsorbent sonicated for 1 hour at 25 °C. 0.1 N HCl and NaOH was used to adjust the pH of the solution. The dosage effect (0.1–0.2 g L^−1^) was optimized by adding adsorbent to 10 ppm TCS solution in 100 mL at the optimized pH. The effect of concentration (10–50 ppm) was performed by adding the optimized adsorbent dosage to different TCS solutions (100 mL) at the optimized pH. The effect of contact time and temperature on the TCS adsorption was further studied at the optimized pH and adsorbent dosage in different kinetic and isotherm models.

#### 2.4.3. Zero-point charge (pH_ZPC_) analysis

The salt addition method was used to calculate the surface charge of the adsorbent as a function of pH.^14,24^ At the zero-point charge/isoelectric point, the net charge of the material becomes zero. The adsorbent solutions in 0.1 M NaCl solution with varying pH^2–12^ were agitated at 150 rpm for 24 hours at 25 °C. The solution was filtered, and the final pH was determined. The ΔpH (difference between the final and initial pH) was plotted against the initial pH to obtain pH_ZPC_.

### 2.5 DFT studies

The experimental results were validated by detailed computational calculations, which provided a solid theoretical foundation for our findings. A truncated polymer (triazine core linked with three carbohydrazide units) with peripheral atoms replaced with hydrogen was considered for the calculations. Both the truncated structure and TCS were optimized to equilibrium geometry using the M06-2x/6-31+G(d,p) theory, confirmed with real frequencies for minimum configurations. Electrostatic potential analysis (ESP) was carried out on the polymer and TCS to understand the electronic distribution, which is important in identifying the possible adsorption sites. Based on these results, TCS can be incorporated into the polymer, and binding energy can be calculated. The different non-covalent interactions resulting in binding can be visualized and analyzed from the scatter plot. All the calculations were performed using Gaussian 09 software.Binding energy = *E*_poly-TCS_ − (*E*_poly_ + *E*_TCS_)*E*_poly_, *E*_TCS_, and *E*_poly-TCS_ are the total energy of the truncated polymer, TCS, and TCS-bound polymer.

## 3. Results and discussions

### 3.1 Synthesis and characterization of CCCH CTP

**Fig. 3:**
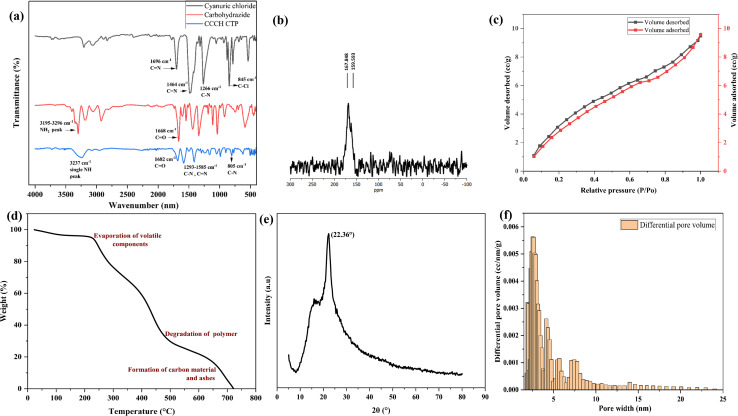
(a) FT-IR spectrum for reactants and polymer CCCH CTP, (b) ^13^C CPMAS NMR spectrum, (c) nitrogen sorption isotherm, (d) TGA curve, (e) XRD pattern, (f) pore size distribution curve for CCCH CTP.

CCCH CTP was synthesized by the condensation reaction between cyanuric chloride and carbohydrazide, as in Fig. 2. The polymer is insoluble in water and other common organic solvents (dimethyl sulfoxide, *N*,*N*-dimethyl formamide, tetrahydrofuran). The initial confirmation for the polymerization of CCCH CTP was analyzed from the ATR-FTIR spectrum (Fig. 3a). The absence of a C–Cl group around 845 cm^−1^ and a single NH stretching peak around 3237 cm^−1^ in the product indicates the substitution of chlorine by the amine group.^25,26^ It directed us to conduct an in-depth study of FT-IR compared to the reactants. The characteristic NH_2_ peak in the carbohydrazide disappeared (3195 to 3296 cm^−1^), whereas a carbonyl peak around 1668 cm^−1^ and triazine peak around 1293 to 1696 cm^−1^ with the breathing mode (805 cm^−1^) is retained during the reaction.^27^ This concludes the complete consumption of building blocks resulting in porous polymer through hydrazide linkages.

**Fig. 4:**
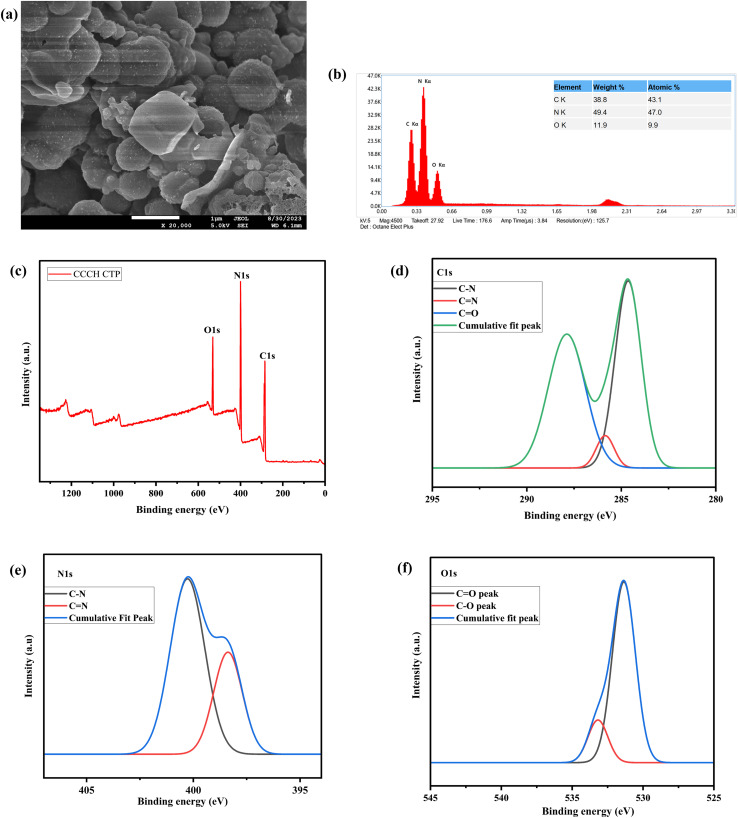
(a) SEM micrographs, (b) EDS spectrum of CCCH CTP, (c) XPS spectrum of CCCH CTP (d) XPS of C 1s spectrum, (e) XPS spectrum of N 1s, (f) XPS spectrum of O 1s.

The chemical structure was further confirmed using ^13^C solid-state NMR (Fig. 3b) and XPS analysis (Fig. S1). The peak around 167 ppm and 159 ppm indicates the presence of carbonyl carbon^28^ and triazine carbon,^29^ respectively. This indicates that the condensation polymerization was successful. The complete XPS spectrum of CCCH CTP revealed the presence of carbon (C) at 285.08 eV, nitrogen (N) at 400.08 eV, and oxygen (O) at 531.08 eV within the framework (Fig. 4c). The C 1s spectrum was further deconvoluted (Fig. 4d) to identify three additional peaks corresponding to C=N at 285.85 eV, C–N at 284.63 eV, and C=O at 287.88 eV functional groups.^25,30^ The N 1s spectrum (Fig. 4e) exhibited two binding energies at 398.37 eV and 400.27 eV, which correspond to the C=N and C–N groups, respectively.^31^ Additionally, two peaks (Fig. 4f) around 531.35 eV indicate the presence of C=O groups, along with another peak at 533.18 eV, which may correspond to C–O groups in O 1s data.^30,32,33^

The structural properties of CTP, including its crystallinity, were analyzed using XRD techniques (Fig. 3e). The absence of peaks at lower diffraction angles, along with a broad peak in the range of 2*θ* = 15 to 30°, with a maximum at 2*θ* = 25°, indicates the semicrystalline nature.^27,34^ This behavior is typical for kinetically controlled irreversible polymerizations of building blocks having angular strain 35. Such strain hinders the symmetrical formation of the network, leading to a likely layered structure due to π–π stacking of triazine rings.^36^

The morphology of CCCH CTP was described using the FE-SEM technique, along with elemental mapping for the composition and distribution of atoms in the polymer. The SEM images (Fig. 4a) reveal that the particles exhibit an irregular spherical shape, which are clustered together to form a porous, interconnected network.^25,37^ The EDS spectrum (Fig. 4b) showcases distinct peaks that indicate a uniform distribution of the essential elements, such as C, O, and N throughout the material.

TGA results indicated that the polymer maintains its stability up to a temperature of 250 °C, as illustrated in Fig. 3d. Initially, a weight loss of approximately 4% was observed, which may be the release of trapped water and other volatiles present within the polymer matrix.^25^ However, significant changes were noted once the temperature exceeded 250 °C; the triazine core began to degrade, leading to the progressive breakdown and complete decomposition of the polymeric structure.^23,26^

The investigation into the porous characteristics of CCCH CTP was conducted by Brunauer–Emmett–Teller (BET) sorption isotherm along with the NLDFT method. The results revealed that the polymer exhibited a reversible type-III sorption behavior (Fig. 3c), characterized by a hysteresis loop, indicating that it behaves as a predominantly mesoporous material.^23,24,31^ The surface area of the polymer was 24 m^2^ g^−1^, with a porous volume of 0.019 cm^3^ g^−1^. Further, the NLDFT method indicated the presence of pores with varying diameters (2–7.4 nm), with the prominent distribution observed at 2.5 nm (Fig. 3f). The comparatively small surface area is likely attributed to the obstruction of pores caused by the presence of functional groups,^14,25,31^ and the random orientation of aggregated polymeric layers, which may impede the accessibility of the pores.

### 3.2 Adsorption of triclosan by CCCH CTP

#### 3.2.1. Optimization of adsorption parameters

The different parameters affecting the adsorption of TCS by the polymer CCCH CTP were investigated and discussed below.

##### 3.2.1.1 Effect of pH

The pH of a solution is crucial in determining how effectively pollutants are adsorbed on the adsorbent surface. It has an impact on the surface charge of the adsorbent and the ionization state of the TCS molecules, influencing potential interactions.

TCS is a highly ionizable compound, with a p*K*_a_ value reported to be 7.9, reflecting its behavior in aqueous environments. When the solution pH is lower than p*K*_a_, the TCS remains undissociated (existing as a molecule).^38–40^ Conversely, when the pH rises above the p*K*_a_, TCS predominantly exists in its anionic, deprotonated state.^2,12,41^ The surface charge of the polymer CCCH CTP was analyzed. According to zero-point analysis, the point of zero charge (pH_ZPC_) is around 5.22, indicating that the adsorbent is acidic in nature (Fig. S1). At pH above the pH_ZPC_, the surface carries negative charges, and *vice versa*. These changes alter the chemical behavior and adsorption characteristics, making pH a crucial factor in the TCS removal from contaminated water sources.

**Fig. 5:**
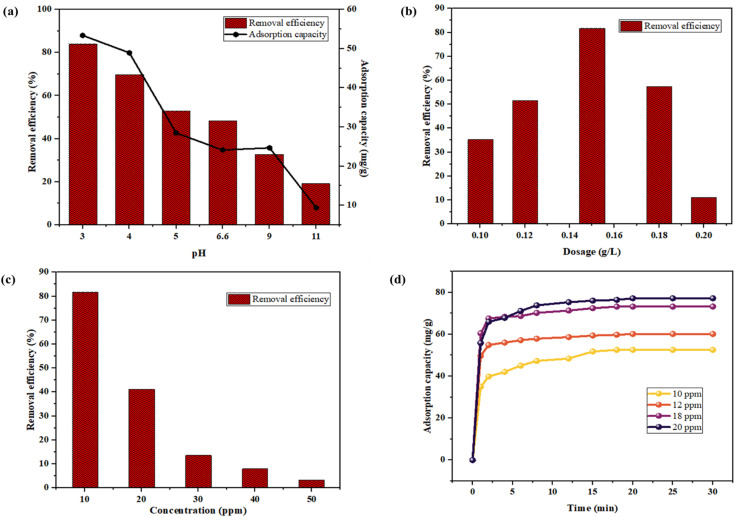
(a) Effect of pH (0.15 g per L CCCH CTP in 10 ppm TCS; 60 min; ultrasound power = 100 W; ultrasound frequency = 40 kHz), (b) dosage (10 ppm TCS; pH = 3; 60 min; ultrasound power = 100 W; ultrasound frequency = 40 kHz), (c) concentration (0.15 g per L CCCH CTP; pH = 3; 60 min; ultrasound power = 100 W; ultrasound frequency = 40 kHz), (d) time (0.15 g per L CCCH CTP in 10 ppm TCS; pH = 3; ultrasound power = 100 W; ultrasound frequency = 40 kHz) on adsorption of TCS by CCCH CTP.

The TCS adsorption by CCCH CTP was compared across the pH range, from acidic to alkaline, with the results indicating a preference for acidic conditions. As the pH increased from 3 to 11, there was a significant decline in the TCS removal efficiency from 84% to 19%, as in Fig. 5a, highlighting that pH 3 is the optimal level for effectively removing TCS. Similar observations were consistent with the literature (Behera *et al.*^38^ and Ma *et al.*^41^). The adsorption capacity of the polymer was reduced from 53 mg g^−1^ to 9 mg g^−1^ with the pH increase. The negatively charged CCCH CTP in the alkaline pH, experiences electrostatic repulsion between the anionic TCS molecules (greater degree of dissociation), hindering adsorption and reducing removal efficiency.^10^ In contrast, undissociated TCS molecules in the acidic medium can alleviate these repulsive interactions with the polymer. Consequently, enhancing the adsorption of TCS, leading to an improved overall removal of TCS.^38,41,42^

##### 3.2.1.2 Effect of dosage

The effect of varying dosage (0.1 g L^−1^ to 0.2 g L^−1^) on the adsorption of TCS was evaluated under acidic conditions, pH 3. The results, as depicted in Fig. 5b, demonstrate that when the dosage increased (0.1 g L^−1^ to 0.15 g L^−1^), the removal efficiency of TCS by CCCH CTP markedly improved from 35.5% to 81.9%. This significant improvement in adsorption can be owed to the availability of additional free binding sites that become accessible with the increased dosage, facilitating greater uptake of TCS up to the maximum at 0.15 g L^−1^.^13^ However, beyond this optimal level to the dosage of 0.2 g L^−1^, the removal efficiency reduced to 11.11%. This reduction in efficiency could be caused by several factors, including the accumulation of particles at higher dosages, which hinders the overall porous area available for adsorption. Additionally, the overlapping of binding sites at elevated dosages may hinder effective adsorption by increasing the diffusion pathway from the surface, thereby limiting the amount of TCS that can be adsorbed.^43,44^ Thus, the dosage of 0.15 g L^−1^ was considered for further studies.

##### 3.2.1.3 Effect of concentration and contact time

Under optimized pH and dosage conditions, the effect of concentration on the TCS adsorption was studied. As the TCS concentration increased from 10 to 50 ppm, removal efficiency decreased from 81.9% to 3.4%, as in Fig. 5c. At lower concentrations of TCS, binding of TCS with available binding sites facilitates a higher rate of adsorption. However, as the concentration increased, TCS molecules in the solution increased, intensifying competition among the molecules for the limited binding sites. This ultimately resulted in a decline in overall adsorption by CCCH CTP.^40^

Each concentration of TCS reaches a state of equilibrium adsorption within a specific time frame. The effect of contact time on adsorption was studied in-depth with initial concentrations from 10 ppm to 20 ppm, as in Fig. 5d. The adsorption capacity showed a significant increase, from 52.5 mg g^−1^ to 77.18 mg g^−1^, with an increase in concentration. Initially, the rate of adsorption was particularly rapid and random due to the high concentration of unadsorbed TCS in the aqueous solution.^10,45^ This facilitated the increased diffusion of TCS molecules due to the available free-binding sites on the adsorbent material.^10,42^ As time progressed and the concentration of free TCS gradually decreased, the system approached a state of saturation, where the equilibrium was attained within 25 minutes. This shift marked a significant change in adsorption dynamics, as fewer TCS molecules were available in the aqueous phase to occupy the binding sites.

#### 3.2.2. Effect of adsorption method

The adsorption technique primarily involves the mass transfer of TCS from the aqueous solution to the polymeric adsorbent across the solid–liquid interface. This process is mainly initiated by conventional shaking or an innovative ultrasound method. Fig. S2 illustrates the impact of these methods on TCS adsorption by CCCH CTP, indicating that ultrasound-assisted adsorption is superior to the shaking method (59% TCS removal and 40.21 mg g^−1^ adsorption capacity). The ultrasound method significantly reduces the equilibrium saturation time to 25 minutes, compared to the conventional shaking method (achieved by 120 minutes). In the shaking method, mass transfer occurs due to turbulence created by mechanical dispersion.^46^ In contrast, the ultrasound method not only relies on mechanical dispersion but also enhances mass transfer through the cavitation effect, which generates high-temperature and high-pressure conditions.^47^ This process exposes the binding sites, improves surface properties,^46^ and breaks up particle aggregation, allowing for quicker adsorption and achieving saturation more rapidly.^47^

#### 3.2.3. Adsorption kinetics

**Fig. 6:**
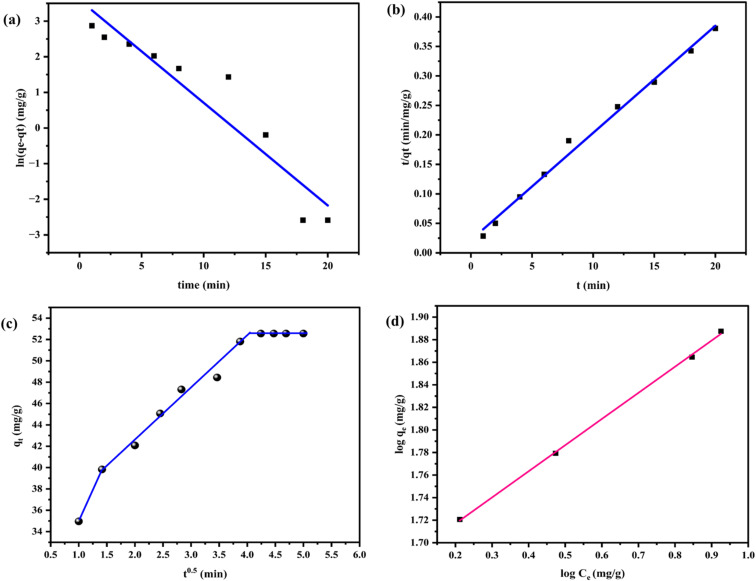
(a) Linear PFO model, (b) linear PSO model, (c) IPD model, (d) Freundlich isotherm for the adsorption of TCS (0.15 g per L CCCH CTP in 10 ppm TCS; pH = 3; 30 min; ultrasound power = 100 W; ultrasound frequency = 40 kHz).

The TCS adsorption as a function of contact time can be explained by well-known kinetic equations of pseudo models of first and second order (PFO and PSO, respectively) along with intra-particle diffusion (IPD) models, which provide valuable insights into the rate and mechanisms of the adsorption process.^13^ A detailed table (Table 1) summarizes several linear kinetic models along with their corresponding equations, kinetic parameters, and correlation coefficients, facilitating a thorough comparison of their effectiveness (taking 10 ppm as an example). Additionally, the figure illustrates the fitting of the experimental data with these different models (Fig. 6a–c), a clear visual representation of the adsorption behavior. The best-fit model is decided by the correlation coefficient (*R*^2^) with the lowest *χ*^2^ value.^48^

**Table 1:** Various kinetic models with their parameters

Kinetic models	Linear equations	Kinetic parameters
PFO	ln(*q*_e_ − *q*_*t*_) = ln *q*_e_ − *k*_f_*t*	*k*_f_ (min^−1^) = 0.288
		*q*_e_ (mg g^−1^) = 36.369
		*R* ^2^ = 0.8831
		*χ* ^2^ = 7.270
PSO	*t*/*q*_*t*_ = 1/*k*_s_*q*_e_^2^ + *t*/*q*_e_	*k*_s_ (g mg^−1^ min^−1^) = 0.01524
		*q*_e_ (mg g^−1^) = 55.035
		*R* ^2^ = 0.9923
		*χ* ^2^ = 0.105
IPD	*q*_*t*_ = *k*_id_*t*^1/2^ + *C*	*k*_id_ (mg g^−1^) min = 4.866
		*C* (mg g^−1^) = 32.145
		*R* ^2^ = 0.9595
		*χ* ^2^ = 0.2617

The TCS adsorption by CCCH CTP follows a pseudo-second-order kinetics (PSO) for all the initial concentrations (10–20 ppm) of TCS.^9,10,45^ This indicates that chemisorption is the rate-determining step, suggesting chemical interactions between TCS and the polymer by sharing electrons.^49^ The calculated qe value of 55.03 mg g^−1^ obtained from the PSO model is consistent with the experimental qe value of 56.79 mg g^−1^ with *χ*^2^ value of 0.105, making this model particularly suitable for describing the kinetics of the TCS adsorption.

The importance of diffusion during the TCS adsorption was investigated using IPD model,^9^ which occurs in three interconnected stages. First, the external mass transfer takes place, where molecules diffuse from the bulk aqueous solution through the boundary interphase to the CCCH CTP surface. Following this, pore diffusion occurs, where the adsorbate penetrates the internal structure of the adsorbent, traversing through its intricate pore network. Ultimately, adsorption leads to the establishment of an equilibrium.^39,40,45^ The linear plots of *q*_*t*_*vs. t*^0.5^ deviate from the origin, suggesting that the rate-controlling step in the adsorption of TCS is influenced by the additional interactions (van der Waals, hydrophobic or π–π stacking) between adsorbent and adsorbate (not by pore diffusion alone).^45^ These findings align with the PSO model, which suggests that rapid initial adsorption is primarily due to various chemical interactions that bind TCS to the adsorbent surface.^9^

#### 3.2.4. Adsorption isotherm

Adsorption isotherm provides valuable insights into the equilibrium distribution of the TCS in the solid–liquid interphase, underlying the plausible mechanism.^9,50^ The experimental data was fitted with different linear isotherm models, including Langmuir (single-layer and homogenous binding sites), Freundlich (multilayer and heterogeneous binding sites), and Temkin isotherm models.^51–53^ The isotherm parameters and correlation coefficient are tabulated, providing an inference on the better fit of the isotherm model (Table S1). The adsorption agreed with the Freundlich model (Fig. 6d), as multiple interactions (H bonding, π–π interactions) favor the binding.^12,13,45^ The polymeric surface is enriched with heterogeneous binding sites with varying energy, allowing cumulative adsorption in multilayers followed by saturation. The maximum adsorbing capacity of the polymer CCCH CTP was calculated to be 83.89 mg g^−1^ and is compared with existing materials along with the adsorption method, summarized in the table (Table S4). Thus, TCS adsorption occurs through a multilayer chemical adsorption mechanism.

#### 3.2.5. Adsorption thermodynamics

In thermodynamics, adsorption can be described as the transfer of adsorbate from its solution across the solid–liquid interface into of the adsorbent. The key parameters influencing adsorption include entropy (Δ*S*°, which acts as the driving force), enthalpy (Δ*H*°), and free energy (Δ*G*°) under standard conditions.^40^ They can be evaluated by the following eqn (3)–(5) and are reported in Table S2:3
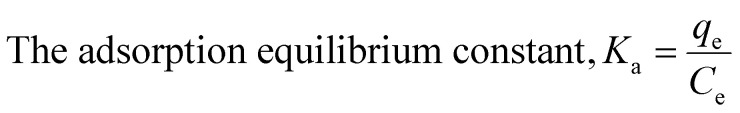

4 Δ*G*° = −*RT* ln(*K*_a_)

5
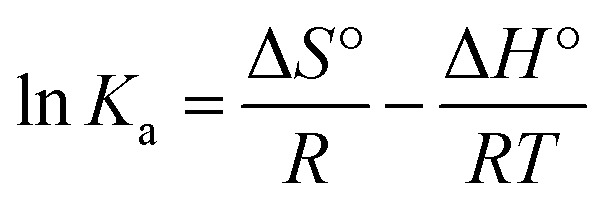
The Δ*H* and Δ*S*° are calculated using the Van't Hoff plot, ln *K*_a_*vs.* 1/*T*, as in Fig. S3.

The negative value for Gibb's free energy is a strong indicator of the thermodynamic spontaneity and feasibility of the TCS adsorption.^9^ As the temperature rises, Δ*G*° becomes increasingly negative,^40^ which reflects the effectiveness of adsorption even at elevated temperatures.^11^ This phenomenon contributes to the increased mobility of the TCS at higher temperatures, facilitating a better interaction between the adsorbate and the adsorbent, ultimately leading to improved adsorption kinetics. The endothermic nature of adsorption can be confirmed by the positive value of enthalpy.^40^ An endothermic reaction absorbs heat from the surrounding environment,^50^ which implies that as temperature increases, the viscosity of the adsorbate solution significantly decreases. This reduction in viscosity promotes a higher diffusion rate for the adsorbate,^50^ enabling it to effectively traverse through the interphase layer to access the binding sites on the CCCH CTP.^9,11^ A positive entropy value (Δ*S*°) signifies an increase in disorder or randomness of the TCS molecules once they are adsorbed onto the surface of the adsorbent compared to the solution phase.^9,40^ This increased randomness is critical, as it promotes significant transport phenomena at the solid–solution interface, facilitating a more efficient and dynamic TCS adsorption.^50^

#### 3.2.6. Possible adsorption mechanism

**Fig. 7:**
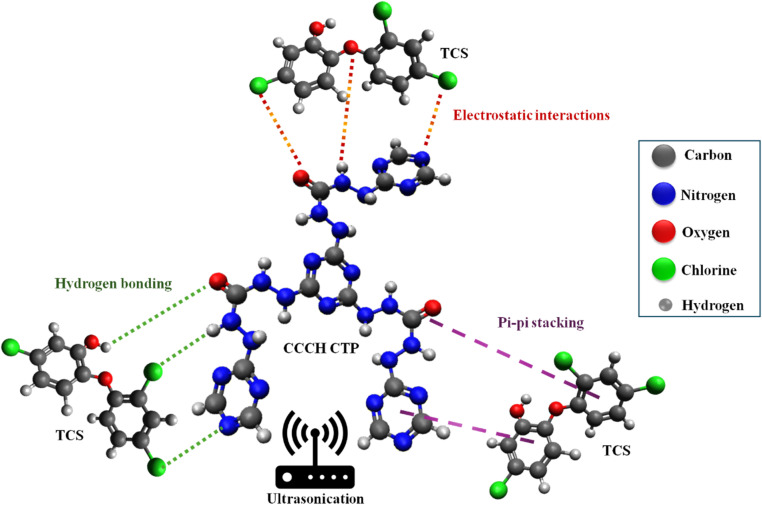
Different possible interactions between CCCH CTP and triclosan.

Electrostatic interactions, π–π interactions, and hydrogen bonding are pivotal in the TCS adsorption by CCCH CTP (Fig. 7). With a dissociation constant (p*K*_a_) of approximately 7.9, triclosan's behavior in solution with the surface charge of the polymer determines the possible electrostatic interactions for adsorption.^2^

**Fig. 8:**
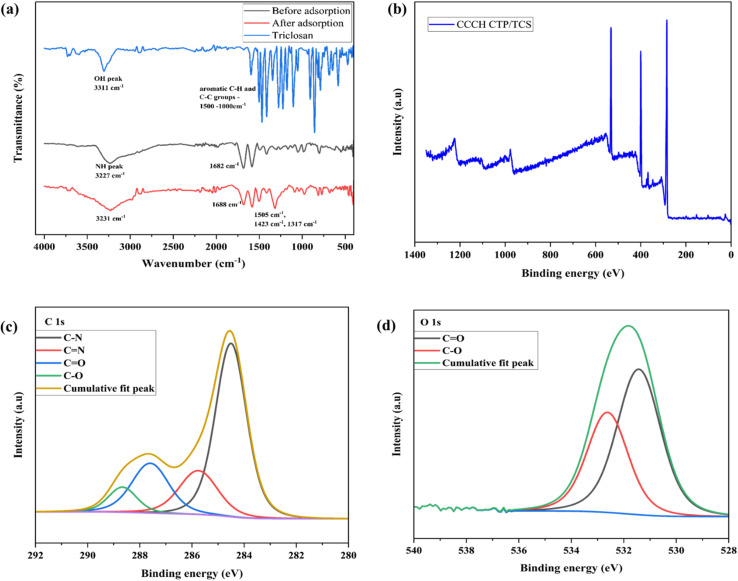
(a) FT-IR plots of TCS and its adsorption by CCCH CTP, (b) XPS spectrum of CCCH CTP/TCS, (c) C 1s spectrum of CCCH CTP/TCS, (d) O 1s spectrum of CCCH CTP/TCS.

Triclosan itself contains aromatic benzene rings with chloro and hydroxy substituents, which enhance its reactivity and interaction capabilities. Simultaneously, the polymer CCCH CTP is rich in triazine rings and comprises basic functional derivatives such as –(C=O) and –(NH) groups, promoting a diverse range of intermolecular interactions. These interactions can be further analyzed using FTIR and XPS data, illustrated in Fig. 8. The FTIR spectrum of triclosan, polymer, and polymer post-adsorption reveals significant spectral features (Fig. 8a). TCS molecules exhibit characteristic peaks, including a weak band associated with C–Cl vibrations around 700 cm^−1^, an O–H stretching at 3311 cm^−1^, and distinct aromatic CH bending and C–C stretching vibrations ranging between 1500-1200 cm^−1^.^54^ Following the adsorption of TCS onto the polymer, a prominent broad band appears around 3231 cm^−1^, indicative of hydrogen bonding interactions by the hydroxyl group of triclosan and the –(NH) groups^55^ or carbonyl oxygen of the polymer, with specific carbonyl peaks shifting slightly from 1682 cm^−1^ to 1689 cm^−1^.^11,56^ Peaks around 1505, 1423, and 1317 cm^−1^ in the spectrum indicate the presence of aromatic stretching of C–C and C–H in TCS. The π electrons residing in the benzene rings of triclosan have the capacity to interact with the triazine rings and carbonyl bonds within CCCH CTP through π–π stacking,^9,10^ contributing to the overall stability and effectiveness of the adsorption process.

The high-resolution XPS spectrum provides insight into potential changes in the electronic states of the elements after the TCS adsorption. An increase in the intensity of the C and O peaks in the overall spectrum indicates the presence of TCS molecules adsorbed to the polymer (Fig. 8b). In the deconvoluted C 1s spectrum (Fig. 8c), there is a decrease of C=O peak intensity, suggesting a possible interaction of TCS with the carbonyl group, along with the appearance of an additional peak at 288.66 eV, indicating the presence of carbon in an electronegative environment, potentially from TCS.^57,58^ Furthermore, a peak at 532.62 eV was identified (Fig. 8d), indicating the presence of an oxygen atom from the –OH group of TCS.^9,58^ The N 1s spectrum did not show any significant changes during the adsorption process (Fig. S4), which may be due to the absence of these elements on the surface or the overlapping of these peaks. These findings suggest possible hydrogen bonding between the –OH group of TCS and the C=O or NH groups, which is consistent with the results from FTIR.^58^

In addition to these interactions, ultrasound-assisted adsorption significantly contributed to achieving equilibrium in a short period.^22^ This was made possible by the enhanced diffusion of TCS, which allowed for better accessibility to the exposed binding sites. The ultrasound energy increased the movement of TCS molecules, optimizing their adsorption onto available binding sites and thereby speeding up the overall process.^21^

### 3.3 DFT studies

**Fig. 9:**
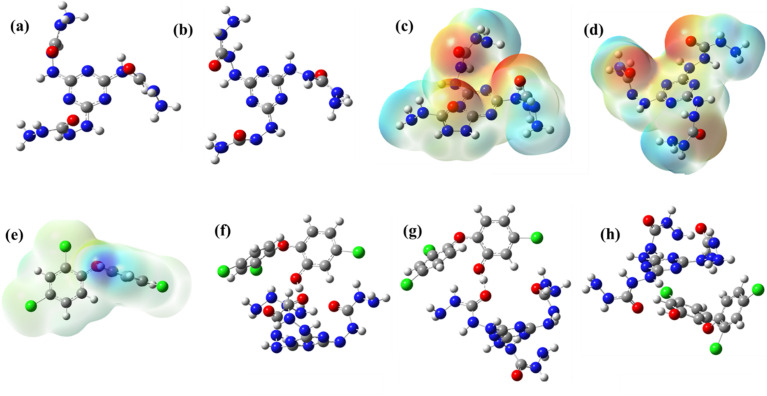
(a) Optimized structure of conformer 1, (b) optimized structure of conformer 2, (c) ESP of conformer 1, (d) ESP of conformer 2, (e) ESP of TCS, (f) optimized structure of Complex 1, (g) optimized structure of Complex 2, (h) optimized structure of Complex 3.

The truncated polymeric structure can exist in two conformations. In conformer 1, all three –(C=O)–NH groups (carbohydrazide) are positioned above the triazine ring. In conformer 2, two of these groups are above the triazine ring, while the other group is located below the plane as in Fig. 9(a) and (b). Both the conformers, along with TCS, are optimized^59^ for further calculations with energy calculated in Table S3. The stabilities of the two conformers are nearly identical, with conformer 2 slightly favored over the other by 0.981 kcal mol^−1^.

The ESP plots of conformers provide the possible binding sites for TCS, where red and blue regions represent electron-deficient and electron-rich regions as in Fig. 9(c)–(e). The mapping indicates electron density accumulation at the –C(=O)–NH regions (in the polymer), while the positive charge is accumulated at the hydroxyl region of TCS.^60^ This prompted us to design the polymer-triclosan in a way that ensures electrostatic interactions between polymer and triclosan. This resulted in Complex 1 (conformer 1 and TCS), Complex 2 (conformer 2, two –(C=O)–NH above the triazine interacts with TCS), and Complex 3 (conformer 2, –(C=O)–NH below the triazine interacts with TCS). The computed geometries of the complexes are given in Fig. 9(f)–(h). Complex 1 is found to be highly favorable, with more negative binding energy value of −27.01 kcal mol^−1^. The high stability of this complex over the complexes can be attributed to the more favorable interaction in the former. We have taken a truncated polymer for constructing the complex with triclosan for calculations. The limitations of the truncation include the ambiguity in selecting the truncation size, which brings in reasonable error in calculations and long-range interactions. The computed binding energy using the truncated model neglects the pore confinement and periodicity effects as well.

Frontier molecular orbital of the stable complex indicates that both HOMO and LUMO have their major contribution coming from the triclosan part. HOMO has small contributions from the polymer, especially the orbitals on carbonyl oxygen and –NH groups (Fig. S5).

**Fig. 10:**
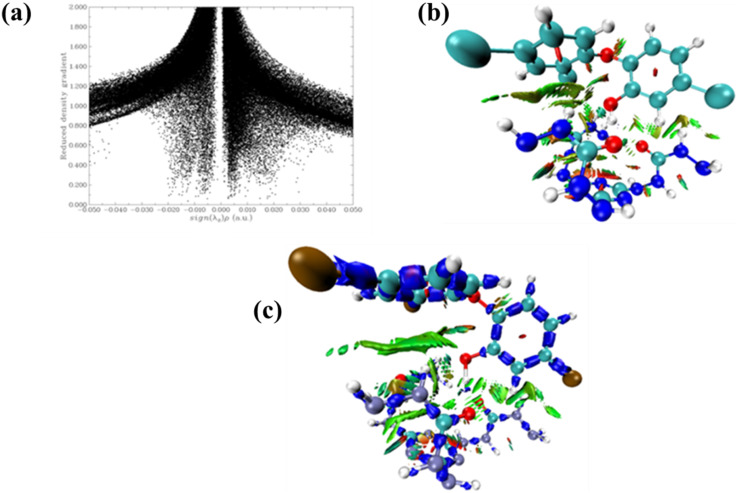
(a) Reduced density gradient (RDG) scatter plot, (b) color-filled RDG plot, and (c) interaction region indicator analysis (IRI).

Scatter plots generated by the analysis of non-covalent interactions on the complexes indicate many van der Waals interactions between the polymer and triclosan. This is consistent with the color-filled RDG plot, where similar interactions are indicated by the green region lying between the polymer and triclosan, along with some steric interactions within its geometry, as indicated by the red region.^61,62^ Interaction region indicator (IRI) analysis also provides the same result: Favorable bonding interactions shown by blue colors, favorable van der Waals interactions by green color, and steric effects by red color (Fig. 10).

## 4. Conclusions

A novel triazine-linked porous polymer was synthesized through the base-catalyzed condensation reaction of cyanuric chloride and carbohydrazide. These stable, nitrogen-rich polymers were characterized using various advanced analytical techniques to validate their structural integrity and composition. Remarkably, these polymers exhibit a porous area of 24 m^2^ g^−1^, complemented by a pore size of 2.5 nm, which enhances their ability to interact with targeted pollutants. In an exciting exploration of their practical application, these heteroatom-rich polymers were investigated for their efficacy in water purification, serving as a sonoadsorbent to effectively remove triclosan – an emerging environmental contaminant notorious for its persistence in aquatic ecosystems. The process of ultrasonication, which initiates acoustic cavitation, facilitates the rapid diffusion and transport of these pollutants toward the extensive surface of the polymer. The optimization of adsorption parameters revealed that equilibrium could be achieved in a remarkably short time of 25 minutes, yielding a removal efficiency of 81%. The adsorption kinetics aligned with PSO dynamics, while the interaction between the triclosan and the polymer conformed to the Freundlich model. Notably, the maximum adsorbing capacity of the polymer was determined to be 83.89 mg g^−1^ under acidic conditions at a pH of 3. The adsorption process is characterized by multilayer chemisorption that engages a variety of interactions, including electrostatic forces, hydrogen bonding, and π–π interactions, which collectively enhance the material's ability to capture pollutants. Additionally, DFT studies performed were consistent with experimental results, reflecting the adsorption ability of CCCH CTP for TCS molecules. This pioneering research illuminates the promising applicability of ultrasound-assisted adsorption techniques for environmental remediation and paves the way for future advancements. The higher adsorption efficiency of simulated TCS wastewater suggests that the polymeric porous adsorbent may effectively remove other emerging pollutants from industrial effluents in a real matrix. Furthermore, potential modifications to the polymer, particularly the incorporation of magnetic properties, could significantly improve the ease of separation and enhance the reusability of these polymeric adsorbents, presenting a forward-thinking approach to sustainable water treatment solutions.

## Conflicts of interest

All the authors declare that they do not have conflicts of interest.

## Supplementary Material

RA-015-D5RA02743H-s001

## Data Availability

The experimental data supporting the research work have been included as part of the SI. See DOI: https://doi.org/10.1039/d5ra02743h.
