# Supplementary material for: Encapturing triclosan from water using a novel sonoadsorbent triazine polymer with carbohydrazide linkages: experimental and theoretical studies
Source: RSC Adv. 2025 Sep 15;15(40):33466–79. doi: 10.1039/d5ra02743h (PMC12434462; doi:10.1039/d5ra02743h)
Supplement: RA-015-D5RA02743H-s001 [file RA-015-D5RA02743H-s001.pdf]

Supplementary Information (SI) for RSC Advances.
This journal is © The Royal Society of Chemistry 2025

Supplementary Information

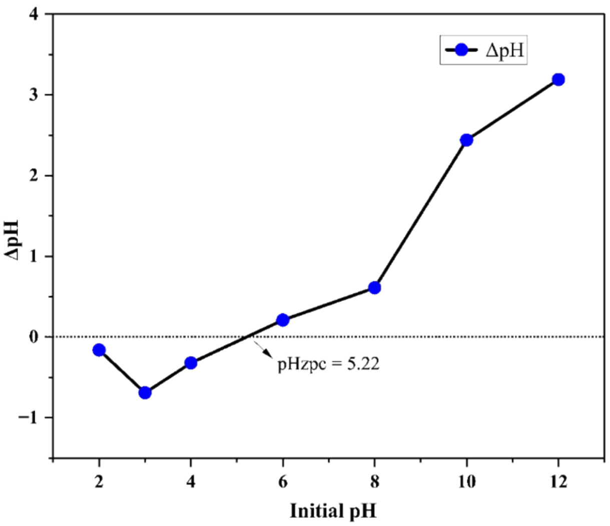

Fig.S1. zero-point charge analysis of CCCH CTP.

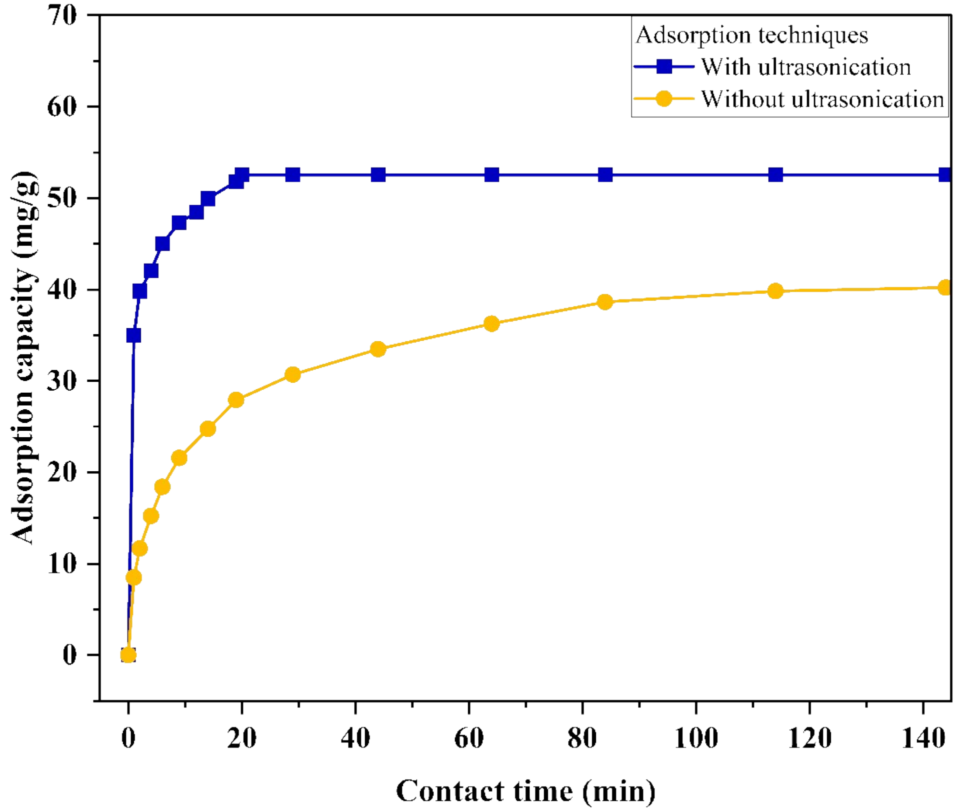

Fig.S2. Comparison of the adsorption method on TCS removal by CCCH CTP.

With ultrasonication (0.15 g/L CCCH CTP in 10 ppm TCS; pH=3; ultrasound power = 100W; ultrasound frequency= 40KHz) and Without ultrasonication (0.15 g/L CCCH CTP in 10 ppm TCS; pH=3; stirring at 180 rpm).

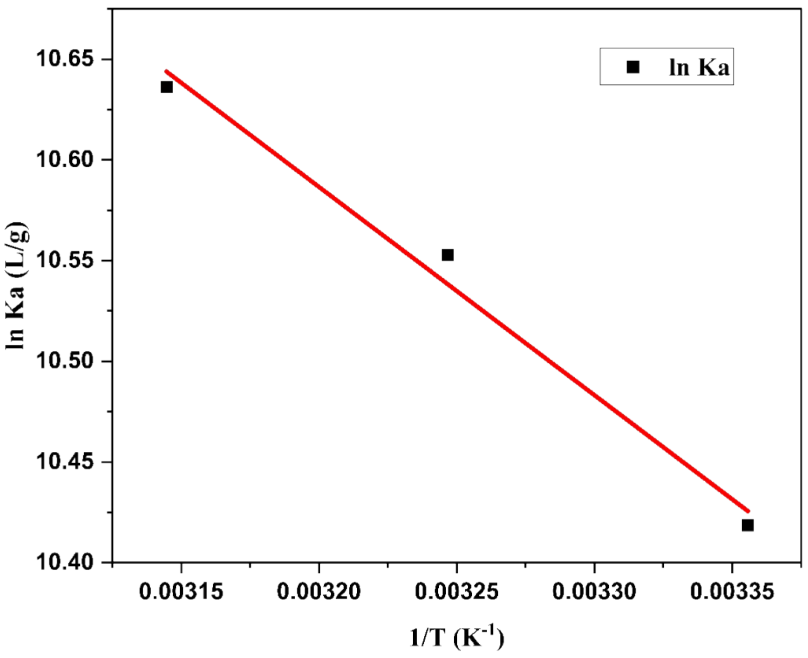

Fig.S3. Thermodynamic plot for the adsorption of TCS.

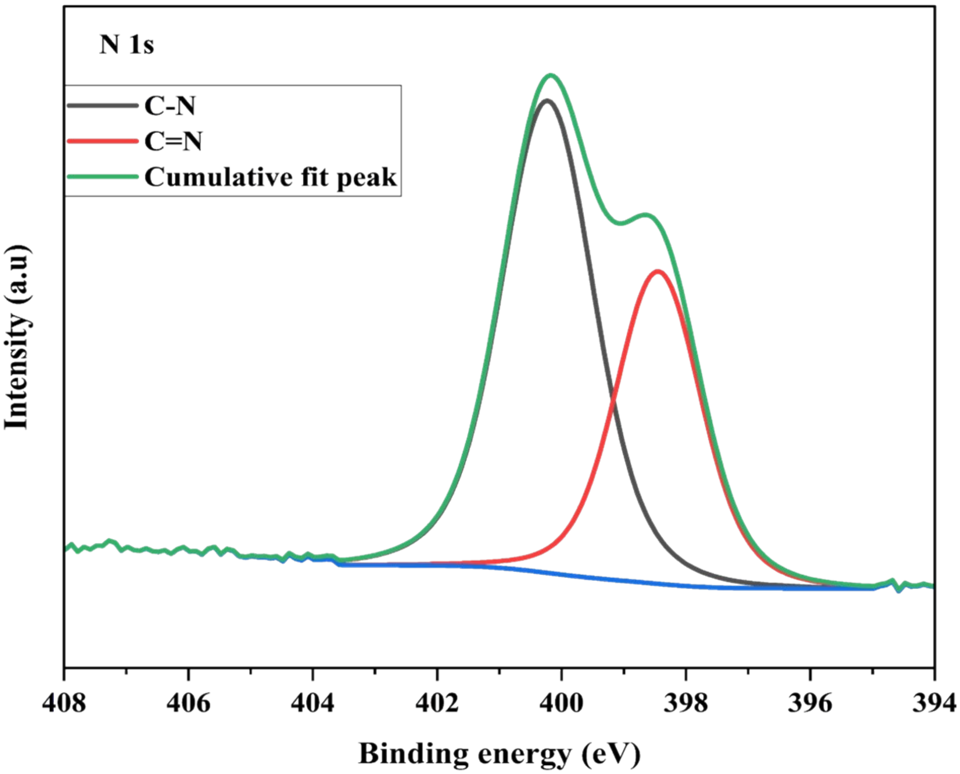

Fig.S4. N1s spectrum of CCCH CTP/TCS.

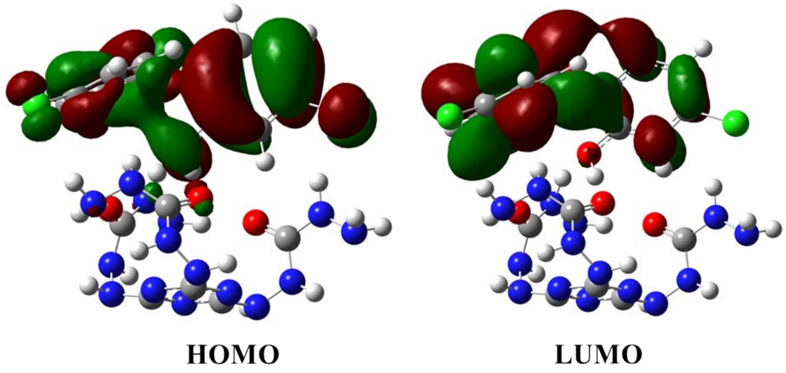

Fig S5: Computed frontier molecular orbitals of Complex 1.

Table S1: Isotherm parameters for triclosan adsorption on the adsorbent.

Isotherm	Equations	Parameters
**Langmuir model**	$\frac{1}{q_e} = \frac{1}{k_L q_m} \cdot \frac{1}{C_e} + \frac{1}{q_m}$	**q_m (mg/g) = 83.89**
		**k_L (L/mg) = 0.989**
		**R^2 = 0.957**
		**χ^2 =84.00**
**Freundlich model**	$q_e =$ [illegible]	**K_F (mg/g) (mg/L)$^{1/n}$ = 46.83**
		**n = 4.31**
		**R^2 = 0.998**
		**χ^2 = 30.185**
**Temkin model**	$q_e = B_T \ln A +$ [illegible]	**B_T = 14.87**
		**A (L/mg) = 20.24**
		**R^2 = 0.993**
		**χ^2 =30.392**

Table S2: Thermodynamic parameters for triclosan adsorption on the adsorbent.

**Temperature (K)**	**Thermodynamic parameters**		
	**ΔG° (kJ/mol)**	**ΔH° (kJ/mol)**	**ΔS° (J/mol K)**
298	-25.813	21.467	89.019
308	-27.023		
318	-28.121		

Table S3: Energies of optimized structures.

**Structure**	**Energy**	**Unit**
Conformer 1	-1284.1269	Energy in Hartree (H) (1H = 627.51 kcal/mol)
Conformer 2	-1284.1284	
TCS	-1992.2082	
Complex 1	-27.01	Binding energy in kcal/mol
Complex 2	-20.24	
Complex 3	-22.08	

Table S4: Comparison of various adsorbents for adsorption of triclosan.

Sl.No	Adsorbent	Adsorption method	q_{max} (mg/g)	Reference
1	Activated carbon derived from coconut pulp	Shaking	2.02	(1)
2	Montmorillonite	Shaking	3.3	(2)
3	Polyvinyl chloride	Shaking	8.98-12.7	(3)
4	F400 (Activated Carbon)	Magnetic stirring	15.5	(4)
5	Norit (Activated Carbon)	Magnetic stirring	16	(4)
6	Ceramic shell-protected granular activated carbon	Shaking	16.54	(5)
7	Darco (Activated carbon)	Magnetic stirring	18.5	(4)
8	Carbon black	Shaking	18.62	(6)
9	Kaolinite	Shaking	22.02	(2)
10	Fe_3O_4 doped hydroxyl functionalized microporous organic polymer (Fe_3O_4/Hy-MOP)	Ultrasonication	26.24	(7)
11	SWCNT	Shaking	30.3	(8)
12	Activated carbon	Shaking	41.15	(2)
13	KNF-750	Shaking	77.4	(9)
14	Zeolites	Shaking	82.25	(10)
15	CCCH CTP	Ultrasonication	83.39	**This work**

References

1. Mohd Khori NKE, Hadibarata T, Elshikh MS, Al-Ghamdi AA, Salmiati, Yusop Z. Triclosan removal by adsorption using activated carbon derived from waste biomass: Isotherms and kinetic studies. 2018;65(8):951-9.

2. Behera SK, Oh S-Y, Park H-S. Sorption of triclosan onto activated carbon, kaolinite and montmorillonite: Effects of pH, ionic strength, and humic acid. Journal of Hazardous Materials. 2010;179(1):684-91.

3. Ma J, Zhao J, Zhu Z, Li L, Yu F. Effect of microplastic size on the adsorption behavior and mechanism of triclosan on polyvinyl chloride. Environmental Pollution. 2019;254:113104.

4. Medellín-Castillo NA, González-Fernández LA, Ocampo-Pérez R, Leyva-Ramos R, Luiz-Dotto G, Flores-Ramírez R, et al. Efficient removal of triclosan from water through

activated carbon adsorption and photodegradation processes. Environmental Research. 2024;246:118162.

5. Ndagijimana P, Liu X, Li Z, Yu G, Wang Y. The synthesis strategy to enhance the performance and cyclic utilization of granulated activated carbon-based sorbent for bisphenol A and triclosan removal. Environmental Science and Pollution Research. 2020;27(13):15758-71.

6. Wang J, Man H, Sun L, Zang S. Carbon Black: A Good Adsorbent for Triclosan Removal from Water. 2022;14(4):576.

7. Zhang T, Wu H, Naghizadeh M, Zheng Q, Dong S. Magnetic Porous Polymer with −OH Groups as Sorbent for Excellent Extraction and Removal of Personal Care Products from Water. Industrial & Engineering Chemistry Research. 2023;62(22):8873-81.

8. González-Fernández LA, Medellín-Castillo NA, Ocampo-Pérez R, Hernández-Mendoza H, Berber-Mendoza MS, Aldama-Aguilera C. Equilibrium and kinetic modelling of triclosan adsorption on Single-Walled Carbon Nanotubes. Journal of Environmental Chemical Engineering. 2021;9(6):106382.

9. Cho E-J, Kang J-K, Moon J-K, Um B-H, Lee C-G, Jeong S, et al. Removal of triclosan from aqueous solution via adsorption by kenaf-derived biochar: Its adsorption mechanism study via spectroscopic and experimental approaches. Journal of Environmental Chemical Engineering. 2021;9(6):106343.

10. de Souza Gonçalves Proença B, de Souza Antonio R, Cusioli LF, Vieira MF, Bergamasco R, Vieira AMS. Assessment of the natural zeolite adsorption capacity for the removal of triclosan from the aqueous medium. Desalination and Water Treatment. 2023;292:165-75.
